# Overcoming the challenges encountered in CAR-T therapy: latest updates from the 2023 ASH annual conference

**DOI:** 10.3389/fimmu.2024.1389324

**Published:** 2024-04-10

**Authors:** Tingting Zhang, Yicheng Zhang, Jia Wei

**Affiliations:** ^1^ Cancer Center, Shanxi Bethune Hospital, Shanxi Academy of Medical Sciences, Tongji Shanxi Hospital, Third Hospital of Shanxi Medical University, Taiyuan, Shanxi, China; ^2^ Department of Hematology, Tongji Hospital, Tongji Medical College, Huazhong University of Science and Technology, Wuhan, Hubei, China; ^3^ Immunotherapy Research Center for Hematologic Diseases of Hubei Province, Wuhan, Hubei, China; ^4^ Department of Hematology, Shanxi Bethune Hospital, Shanxi Academy of Medical Sciences, Tongji Shanxi Hospital, Third Hospital of Shanxi Medical University, Taiyuan, Shanxi, China; ^5^ Sino-German Joint Oncological Research Laboratory, Shanxi Bethune Hospital, Shanxi Academy of Medical Sciences, Taiyuan, Shanxi, China

**Keywords:** CAR-T, CRISPR Cas-9, combination strategies, biomarker, targets, ASH

## Abstract

Chimeric antigen receptor (CAR) -T cell therapy has entered the breakthrough era, characterized by a blend of therapeutic opportunities and challenges. With the integration of genome-editing technology, CAR-T cells will be empowered to become super warriors in eradicating tumor cells and attacking various tumors, including T-cell malignancies and acute myeloid leukemia. Notably, the optimization of CAR-T cells, including efficacy, safety, and manufacturing speed, coupled with other therapeutic strategies such as radiotherapy, hematopoietic stem cell transplantation, small-molecule inhibitors, and bispecific antibodies, could revolutionize the therapeutic landscape of tumors. Consequently, next-generation cellular immunotherapy, including universal CAR-NK cells and synergistic combination approaches, are anticipated to significantly impact cancer treatment in the coming decade. Nevertheless, the failure rates of CAR-T therapy continue to be significant. The challenge lies in determining the optimal combination strategy and identifying reliable and robust biomarkers to effectively select the patients who will derive the greatest benefit from CAR-T therapy. Herein, we highlight recent innovations in CAR-T products, combination strategies and predictive biomarkers of response presented at the 2023 ASH Annual Meeting.

## Introduction

**Figure 1:**
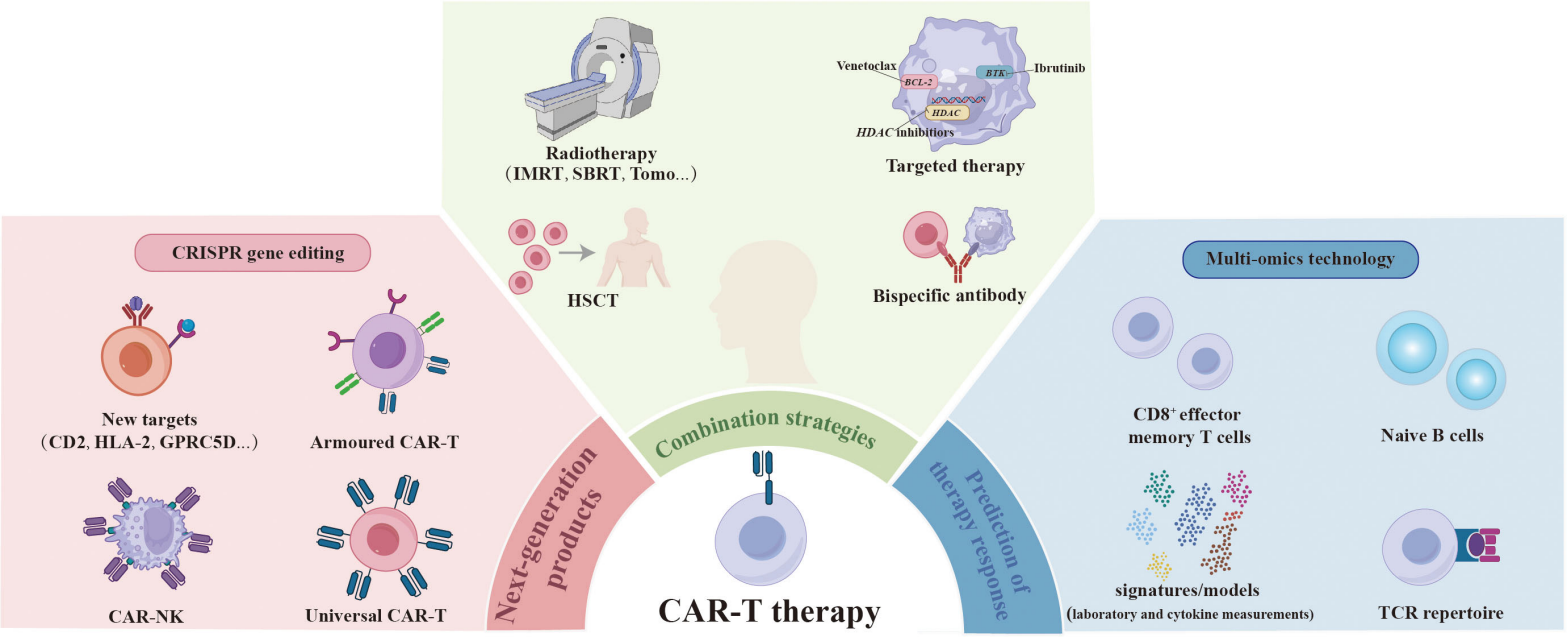
Overcoming the challenges encountered in CAR-T therapy. The next generation of CAR-T products is being engineered to eradicate tumor cells more effectively by designing new targets, as well as developing armored CAR-T and universal CAR-T and CAR-NK through genome-editing technology. The integration of CAR-T therapy with radiotherapy, HSCT, targeted therapy, and bispecific antibody approaches is expected to further enhance therapeutic efficacy and overcome resistance. Certain pre-treatment attributes have been identified as predictors of response to CAR-T therapy through multi-omics technology, including higher levels of CD8+ effector memory T cells and naïve B cells, greater diversity in the TCR repertoire, and inflammatory biomarker signatures/models that incorporate pre-CAR-T-infusion laboratory and cytokine measurements. CAR, chimeric antigen receptor; HSCT, hematopoietic stem cell transplantation; TCR, T cell receptor.

Chimeric antigen receptor (CAR)-T cell therapy is emerging as a precise, rapid, and efficient approach to cancer treatment (1). In the largest real-world study conducted by the US Lymphoma CAR-T Cell Consortium, 275 patients with relapsed or refractory large B-cell lymphoma (LBCL) who received standard-of-care axicabtagene ciloleucel, exhibited a 5-year progression-free survival (PFS) rate of 28.5% and an overall survival (OS) rate of 40.3%. These results align with those of the ZUMA-1 trial (2), which, for the first time, demonstrated remarkable efficacy in treating B-cell malignancies in a real-world setting (3). Continuous developments in innovative CAR-T products and ongoing exploration of combination treatment strategies aim to enhance the antitumor activity further. The development of biomarkers to predict response to immunotherapies contributes to maximizing treatment efficacy and improving patient survival. This overview summarizes the most exciting advances and innovations in CAR-T products, combination approaches, and predictive biomarkers presented at the 2023 ASH Annual Meeting (**Figure 1**).

## Engineering next-generation CAR-T products

BCMA-targeted CAR-T cell is one of the most representative cellular immunotherapies in multiple myeloma (MM) (4). Numerous preclinical and phase I/II studies have focused on enhancing the persistence and functionality of CAR-T cells. Bachl et al. (5) conducted a genome-wide CRISPR loss-of-function screen to identify key genes that induce T cell dysfunction. Notably, the ablation of CUL5 was found to enhance the persistence and functionality of BCMA and CD19-specific CAR-T cells. By exploring new targets, studies aim to overcome CAR-T resistance. The first-in-human study of BMS-986393, a GPRC5D-targeted autologous CAR-T cell therapy, demonstrated a manageable safety profile (6). Updated results showed that BMS-986393 induced deep and durable responses across all tested dosages in relapsed/refractory MM patients, with a complete response (CR) rate of 46% in patients refractory to prior BCMA-directed therapies (7). This suggests a potential strategy to overcome BCMA CAR-T cell resistance.

Universal CAR is a research hotspot due to its unique advantages, including shorter processing time, lower cost, and increased accessibility. NK cells are considered ideal candidates for developing novel cellular immunotherapies. Duong et al. (8) constructed chimeric Ig-like transcript (ILT) receptors (CIR) containing ILT2 or ILT4 to target HLA-G expressed on AML cells. Of note, CIR-NK cells efficiently eliminated over 90% of HLA-G+ AML cells. Additionally, the inclusion of MyD88 signaling in the CIR constructs significantly enhanced antitumor activity. Meanwhile, Xiang et al. (9) developed an allogeneic ‘universal’ CD2-targeting CAR-T cell by deleting both CD2 and the T-cell receptor α subunit. When combined with rhIL-7-hyFc, a long-acting IL-7, this approach overcame the impaired anti-tumor activity caused by CD2 deficiency and induced durable CR in mouse models of T-cell malignancies.

**Table 1** summarizes the most novel and effective CAR-T products. Overall, the innovation prospects of next-generation of CAR-T cells primarily include: 1) leveraging CRISPR-Cas9 genome editing technology to enhance the intrinsic activity of CAR-T cells; 2) transitioning from autologous CAR-T cells to ‘off-the-shelf’ CAR-T/NK cells; 3) constructing more robust armored CAR-T cells; 4) optimizing manufacturing operations, including time consumption, T-cell sorting, and virus packaging strategies, etc.

**Table 1:** Properties of next-generation CAR-T products presented at the 2023 ASH annual meeting.

Disease	Cell source	Target	Product name	Modifications	Improved methods	Purpose	Type of trial	References
MM	donor T cell	BCMA	–	CUL5 ablation	Genome editing	Efficacy	–	(5)
MM	donor T cell	GPRC5D	BMS-986393	–	New target	Safety and efficacy	clinical	(7)
AML	donor NK cell	HLA-G	CIR-NK cell	CIR (ILT2 or ILT4)	New target	Efficacy	preclinical	(8)
T-cell malignancies	off-the-shelf T cell	CD2	UCART2	CD2 and the TCR-α subunit deletion	Genome editing	Efficacy	preclinical	(9)

AML, acute myeloid leukemia; BCMA, B cell maturation antigen; CAR, chimeric antigen receptor; CIR, chimeric Ig-like transcript receptors; ILT, Ig-like transcript; MM, multiple myeloma, UCART2 ‘universal’ CD2-targeting CAR-T, - not available.

## Optimization of combination treatment strategies

We have previously overviewed combination treatment strategies for overcoming resistance to CAR-T cell therapy (10). Integrating CAR-T therapy into established treatment modalities, including radiotherapy, hematopoietic stem cell transplantation (HSCT), as well as novel targeted therapy and immunotherapy approaches, holds promise in inducing synergistic antitumor effects and improving therapeutic efficacy (11).

Radiotherapy has the potential to remodel the tumor microenvironment and enhance the function of CAR-T cells (12). In a mouse model of LBCL, low-dose total body irradiation at 1 Gy before CAR-T cell administration achieved a high CR rate (13). Clinically, patients with bulky disease may be ideal candidates for this combined approach. However, the timing and dose of radiotherapy remain controversial and require further exploration.

Additionally, a novel combination strategy (14) involves directly bridging to haplo-HSCT after CD7 CAR-T cell infusion in CD7+ acute leukemia patients without using a conditioning regimen or GvHD prophylaxis. At a median follow-up of 10.2 months after CAR-T therapy, 6 of 9 evaluable patients retained the MRD^-^ CR status. Taken together, this strategy could avoid eradicating residual CAR-T cells and minimize treatment-related complications.

Meanwhile, Zhang et al. (15) evaluated the efficacy of CAR-T therapy in combination with dasatinib maintenance therapy in newly diagnosed Ph-positive acute lymphoblastic leukemia (ph+ ALL) patients. With a median follow-up of 13.5 months, 14 out of 18 patients (77.8%) remained in CR. This study presented a highly effective chemo-free treatment strategy with low toxicity.

Moreover, Sesques et al. (16) assessed the effectiveness of glofitamab monotherapy in patients who were previously refractory or showed resistance to CAR-T therapy. In another study, Elizabeth et al. (17) presented findings on the efficacy of mosunetuzumab in combination with polatuzumab vedotin in refractory CAR-T recipients.

**Table 2** summarizes the promising synergistic strategies of CAR-T cells. Selecting the combination approach remains challenging, requiring a careful balance of the pros and cons. The uncertainty regarding the efficacy and the potential increase in toxicity of combination strategies cannot be ignored. Comprehensive consideration is necessary to determine and validate the most appropriate patient population, both in well-designed trials and in a real-world setting.

**Table 2:** Selected studies on the synergistic strategies of CAR-T cells presented at the 2023 ASH annual meeting.

Disease	LBCL	CD7^+^ AL	Ph^+^ ALL	B-NHL	LBCL
**Type of trial**	preclinical	clinical	clinical	clinical	clinical
**Patients**	–	relapsed/refractory; ineligible for allo-HSCT	newly diagnosed	relapsed/refractory	relapsed/refractory
**Prior CAR-T therapy**	No	No	No	Yes	Yes
**Targets or products**	CD19 CAR-T cells	CD7 CAR-T cells	CD19 and CD22 CAR-T cells	axi-cel, tisa-cel, brexu-cel, and investigational CAR T-cell	–
**Combination** **Strategies**	LDTBI	haploidentical HSCT	dasatinib	glofitamab	Mosunetuzumab + polatuzumab vedotin
**Number of participants**	–	10	18	63	35
**Median follow-up time (mo)**	–	10.2	13.5	9.7	18.6
**ORR/CR**	–	MRD- CR: 66.7%	CMR: 77.8%	CMR: 36.4% for DLBCL cohort 52.6% for non-DLBCL cohort	ORR: 60% CR: 45.7%
**PFS/OS**	–	OS: 64.3% PFS: 51.4%	–	mOS:17.6 mo for DLBCL cohort NR for non-DLBCL cohort mPFS: 4.9 mo for DLBCL cohort 4.1 mo for non-DLBCL cohort	–
**Side effects**	–	grade 1-2 CRS (90%); grade 2 aGvHD (20%); grade 4 pancytopenia and bone marrow hypocellularity (100%);	No grade 3 or higher CRS or ICANS	grade 1-2 CRS (14.3%); grade 2 neurologic events (3.2%); grade 3 or above neutropenia (33.3%); infection (27.0%);	–
**References**	(13)	(14)	(15)	(16)	(17)

aGVHD, acute graft-vs-host disease; axi-cel, Axicabtagene ciloleucel; B-NHL, non-Hodgkin B-cell lymphoma; brexu-cel, Brexucabtagene Autoleucel; CAR, chimeric antigen receptor; CD7+ AL, CD7-positive acute leukemia; CMR, complete metabolic response; CR, complete response; CRS, cytokine release syndrome; DLBCL, diffuse large B-cell lymphoma; ICANS, Immune effector cell-associated neurotoxicity syndrome; LBCL, large B-cell lymphoma; LDTBI, low dose total body irradiation; mo, months; mOS, median overall survival; mPFS, median progression-free survival; MRD, measurable residual disease; NR, not reached; ORR, overall response rate; OS, overall survival; PFS, progression-free survival; ph+ ALL, Ph-positive acute lymphoblastic leukemia; tisa-cel, Tislecagenleucel; - not available.

## Determinants of response and resistance to CAR-T therapy

The certain characteristics of CAR-T cells, the tumor, and the tumor microenvironment have been recognized to predict patients’ outcome on CAR-T therapy. Blocking the exhaustion process, including inhibitory signaling pathways, is crucial for maintaining the persistence of CAR-T cells. Multidimensional omics analyses, encompassing genomics, single-cell transcriptomics, proteomics, metabolomics, and T cell receptor-repertoire profiling, provide unique opportunities to dissect the determinants of response and resistance to CAR-T therapy.

Maximilian et al. conducted a longitudinal single-cell multi-omics study to identify factors predicting the response to BCMA-directed CAR-T cells. They observed that patients who achieved CR had notably higher levels of CD8+ effector memory T cells (TEM) at leukapheresis and greater diversity in the T cell receptor (TCR) repertoire over time compared to non-CR patients (18). Another study at the single-cell level, led by Maurer et al., revealed an increased proportion of B cells, predominantly naïve B cells, along with a trended higher lymphocyte to monocyte ratio at baseline in responders compared to non-responders. Additionally, the infusion products exhibited a higher proportion of CD8+ T cells and more significant clonal expansion of CD8+ TEM cells with elevated expression of T cell activation genes in responders compared to non-responders (19).

To improve accessibility, Raj et al. developed the InflaMix model, which can be derived using a simple blood draw and identify a unique inflammatory biomarker signature based on pre-CAR-T-infusion laboratory and cytokine measurements. They found that patients in the inflammatory clusters had a higher risk of not achieving CR in the ZUMA-1 and ZUMA-7 cohorts (20).

Differences between responders and non-responders can be identified at the time of leukapheresis. The implementation of multi-omics technologies can provide new insights into the biology of CAR-T cells and enhance CAR-T cell manufacturing, thereby extending CAR-T cell persistence. Furthermore, these attributes can be utilized to improve patient selection and enable clinicians to predict and monitor patient responses more effectively.

## Conclusion

With the ongoing development of adoptive cellular immunotherapy, integrating more effective CAR-T cells with other therapeutic approaches has significantly expanded the scope of cellular immunotherapies and is envisioned as a potentially curative strategy. The crucial challenge lies in identifying the most effective way to implement this approach and uncover pretreatment biomarkers that predict response to advance immunotherapies. Currently, extensive studies are underway to evaluate superior drugs and determine the optimal strategy to enhance the duration of remission following CAR-T cell therapy.

## Author contributions

TZ: Writing – original draft. YZ: Writing – review & editing. JW: Writing – review & editing.
